# Spider-Leg-Inspired Structural Design and Bézier Foot Trajectory Planning for Stable Walking of a Hexapod Robot

**DOI:** 10.3390/biomimetics11050352

**Published:** 2026-05-20

**Authors:** Jian Wu, Yijing Xiong, Hao Shi, Peng Ning, Zhenfeng Li, Ziyang Xu, Jingxin Zhu, Wenwei Xia

**Affiliations:** 1 School of Mechanical Engineering, Nanjing University of Science and Technology, Nanjing 210094, China; 2 Unit 63969, Dingyuan County, Chuzhou 233200, China; 3 Yangzhou Jinxin Cable Co., Ltd., Gaoyou 225600, China

**Keywords:** hexapod robot, biomimetic leg design, workspace-based leg proportion, Bézier foot trajectory, walking stability

## Abstract

Hexapod robots are attractive for operation in cluttered and uneven environments, but their walking stability is strongly affected by the coupled effects of leg morphology and foot-end trajectory planning. In many existing designs, leg-segment proportions, reachable workspace, and swing-phase trajectory smoothness are considered separately, which makes it difficult to clarify how structural parameters and motion planning jointly influence locomotion stability. To address this issue, this study presents a spider-leg-inspired hexapod robot with a simplified three-degree-of-freedom leg configuration. Selected functional characteristics of spider legs, including segmented limb structure and compliant distal contact, were abstracted into an engineering-feasible hexapod platform rather than directly reproducing spider anatomy. A parametric workspace analysis was conducted under a fixed total leg length to compare six candidate femur-to-tibia ratios. Based on forward reach, vertical foot-lifting capability, stride potential, and structural compactness, a 4:6 femur-to-tibia ratio was selected. In addition, an eleventh-order Bézier curve was developed for swing-phase foot trajectory planning and compared with a conventional composite cycloid trajectory under identical tripod-gait conditions. Simulation and straight-line walking experiments showed that the Bézier-based trajectory reduced body-attitude fluctuation and produced smoother angular-velocity variation than the composite cycloid trajectory. The results indicate that the proposed structural design and Bézier-based trajectory can improve flat-ground walking stability of the hexapod robot. This work provides a practical reference for biomimetic structural design and gait-trajectory optimization of multi-legged robots, while further validation on more complex terrain remains necessary.

## 1. Introduction

Legged robots offer clear advantages over wheeled and tracked systems in cluttered or uneven environments because they can generate discrete footholds, step over obstacles, and adjust body posture to irregular terrain. Among different legged configurations, hexapod robots are especially attractive because they provide a practical balance between locomotion stability, maneuverability, and mechanical complexity. Owing to their redundant support legs and alternating tripod-gait capability, hexapod robots have been widely investigated for exploration, inspection, rescue, and other tasks that require robust mobility in unstructured environments [1,2,3].

Over the past two decades, numerous hexapod robots have been developed to improve mobility and environmental adaptability. Representative examples include the compliant-legged RHex platform [4], lunar-exploration hexapod robots with improved triangular gaits [5], and terrain-interaction-aware hexapod control systems [6]. These studies show that locomotion performance is influenced not only by gait scheduling and control algorithms, but also by the mechanical characteristics of the leg structure itself. Therefore, robot morphology, leg workspace, gait parameters, and foot–ground interaction should be considered together when improving walking stability. Recent studies have further extended hexapod locomotion research toward posture control on irregular terrain and motion planning under multi-dimensional terrain features [7,8].

Biomimetic design provides an effective route for improving legged robots by transferring functionally relevant principles from biological systems to engineering applications. However, the objective of biomimetic robot design is not to reproduce a biological organism literally, but to abstract useful structural or functional characteristics and implement them in a technical system. Although spiders are octopods rather than hexapods, their segmented legs, lightweight exoskeletal architecture, coordinated multi-joint motion, and compliant distal contact provide valuable inspiration for multi-legged robot design [9]. Recent bionic spider robot studies have also shown that directly increasing the number of joints and degrees of freedom can lead to greater mechanical complexity, higher control difficulty, increased energy consumption, and higher fabrication cost [10]. Therefore, for practical engineering implementation, it is necessary to simplify biological limb characteristics into a compact and controllable mechanical structure.

In existing hexapod research, one important line of work focuses on leg mechanism design, segment proportion selection, and compliance at the foot end. Previous studies have suggested that leg-segment geometry, compliant foot-end design, and foot–ground interaction can affect reachable workspace, obstacle-crossing ability, body trajectory, and locomotion accuracy [11,12,13,14]. These findings indicate that structural design should be evaluated not only from the standpoint of mechanism realization, but also in relation to forward reach, vertical foot-lifting capability, stride potential, and walking stability. Nevertheless, in many practical hexapod designs, leg proportions are still selected mainly according to experience or mechanical packaging constraints, and their relationship with workspace characteristics and gait performance is not always quantitatively clarified.

Another major line of research concerns gait generation and foot trajectory planning. Foot-end trajectories determine swing clearance, lift-off smoothness, touchdown transition, support continuity, and ultimately body-attitude fluctuation during walking. Traditional trajectory functions, such as sinusoidal, cycloid, polynomial, and spline-based trajectories, have been widely used in legged robots [15,16]. Recent studies on foot-end trajectory control have also shown that coordinated trajectory regulation can improve body-attitude stability during legged locomotion [17]. The composite cycloid trajectory is particularly common because it provides adjustable stride length and leg-lift height with relatively simple implementation. However, when the foot trajectory is not sufficiently smooth during lift-off and touchdown, impact and body oscillation may still occur. Recent gait-optimization studies have therefore emphasized the need to jointly consider swing-leg trajectory, gait period, duty factor, and stability-related indicators [18,19,20,21]. In this context, Bézier curves are attractive for foot-end trajectory planning because their control points provide intuitive geometric regulation of the swing path and make it possible to adjust lift-off, mid-swing, and touchdown regions separately.

Although structural design and gait planning have both been widely studied, they are often treated as largely separate problems. Relatively few studies establish a clear link between leg-segment proportion, reachable workspace, foot-trajectory smoothness, and experimentally observed walking stability. This limitation makes it difficult to explain how structural parameters and motion planning jointly affect the locomotion performance of a biomimetic hexapod robot.

To address this gap, this study presents a spider-leg-inspired hexapod robot in which selected functional characteristics of spider legs are abstracted into a simplified three-degree-of-freedom leg mechanism suitable for an engineering hexapod platform. The main contributions of this work are as follows. First, a biomimetic design rationale is established by simplifying spider-like segmented leg morphology into a compact 3-DOF mechanical leg, thereby reducing structural and control complexity while retaining the essential functions of leg placement, foot lifting, and distal support. Second, a parametric workspace analysis is conducted to compare different femur-to-tibia ratios under a fixed total leg length, and a 4:6 ratio is selected by considering forward reach, vertical foot-lifting capability, stride potential, and structural compactness. Third, an eleventh-order Bézier curve is introduced for swing-phase foot trajectory planning and compared with a conventional composite cycloid trajectory under identical tripod-gait conditions. Body-attitude angles and angular velocities are used as stability indicators to evaluate the influence of the proposed trajectory on straight-line walking stability. The present study focuses on flat-ground walking and aims to provide a practical reference for biomimetic structural design and gait-trajectory optimization of hexapod robots.

## 2. Materials and Methods

### 2.1. Biomimetic Design Rationale

The present design is described as spider-leg-inspired rather than spider-mimicking because the purpose of this work is not to reproduce spider anatomy literally. Instead, selected functional characteristics of spider legs are abstracted and transferred to a six-legged engineering platform. A biological spider leg usually consists of seven major segments, namely the coxa, trochanter, femur, patella, tibia, metatarsus, and tarsus. These segmented limbs provide coordinated multi-joint motion, a relatively large reachable workspace, lightweight distal support, and compliant interaction with the ground [22]. The relationship between the biological leg terminology and the simplified engineering implementation is further described in Section 2.4.

However, directly reproducing the complete anatomical structure of a spider leg would substantially increase the number of joints, actuators, structural mass, and control variables. Such complexity is not necessary for the main objective of this study, which is to investigate the relationship among leg-segment proportion, foot-end workspace, swing trajectory, and walking stability in a hexapod robot. Therefore, the biological leg morphology was simplified into a three-degree-of-freedom (3-DOF) serial leg architecture. In this simplified architecture, the coxa joint mainly provides horizontal leg placement, whereas the femur and tibia joints generate sagittal-plane lifting and forward swing motion. The distal foot-end module provides compliant support during touchdown.

Although spiders are octopods, the present robot adopts a hexapod configuration because six legs provide sufficient static support for tripod-gait walking while reducing mechanical redundancy and control complexity compared with an eight-legged platform. The spider-inspired element of the design therefore lies in the abstraction of segmented limb morphology, coordinated joint motion, and compliant distal contact, rather than in the direct reproduction of the number of legs or the complete biological anatomy. This biomimetic simplification allows the robot to retain the functional advantages relevant to stable walking while remaining feasible for mechanical fabrication, kinematic modeling, and experimental implementation.

### 2.2. Overall Mechanical Configuration of the Hexapod Robot

**Figure 1:**
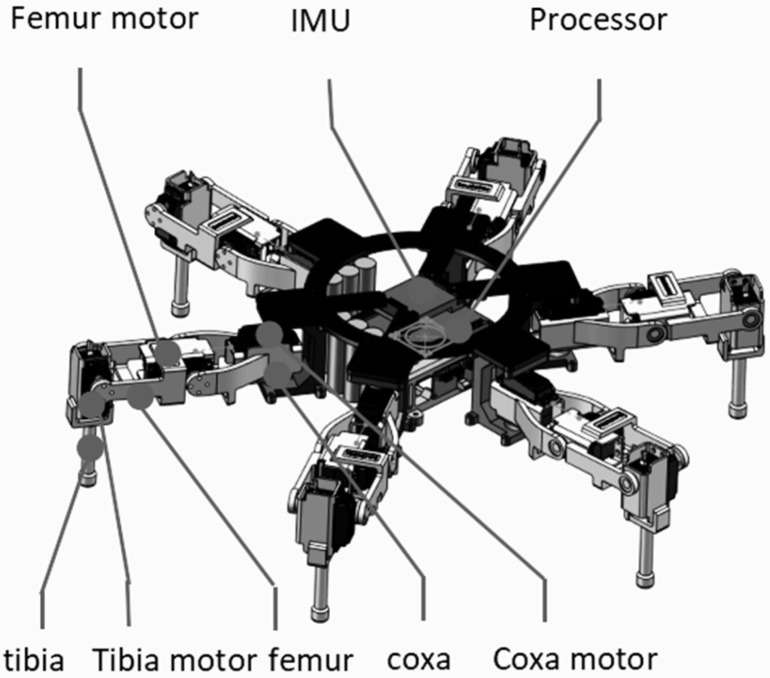
Overall mechanical configuration of the spider-inspired hexapod robot, showing the radially symmetric six-legged layout, central body frame, leg modules, processor, and inertial measurement unit.

Based on the biomimetic design rationale described above, a simplified hexapod robot prototype was developed for evaluating the influence of leg proportion and foot trajectory on walking stability. The robot adopts a radially symmetric layout with six identical legs evenly distributed around a rigid central body, as shown in Figure 1. This configuration was selected because it provides sufficient static support for alternating tripod-gait walking while avoiding the additional mechanical redundancy and control complexity of an eight-legged platform.

Each leg has three active degrees of freedom, corresponding to one proximal yaw motion and two sagittal-plane joint motions. This arrangement enables horizontal leg placement, vertical foot lifting, and forward swing motion, which are the essential functions required for straight-line walking. Because all six legs share the same structural parameters, the mechanical design, kinematic modeling, and control implementation can be simplified. The identical-leg configuration also helps maintain a balanced mass distribution and consistent support characteristics during periodic gait execution.

The central body was designed as a compact load-bearing frame for mounting the leg modules, processor, inertial measurement unit, power supply, and other electronic components. Non-essential biological details were not reproduced, and only the mechanical structure required for leg mounting, load transmission, and experimental walking validation was retained. The resulting prototype has overall dimensions of approximately 550 mm × 550 mm × 240 mm. The symmetric body layout and modular leg arrangement provide a stable mechanical basis for the subsequent workspace analysis, kinematic modeling, and Bézier foot-trajectory experiments.

### 2.3. Leg-Proportion Design Based on Workspace Analysis

To determine the main geometric parameters of the robotic leg, a workspace-based multi-criteria screening method was adopted. The objective of this analysis was not to reproduce the exact segment proportions of a biological spider leg, but to identify a mechanically feasible femur-to-tibia ratio that could provide sufficient forward reach, vertical foot-lifting capability, stride potential, and structural compactness for tripod-gait walking.

The total length of a single leg was fixed at 250 mm according to the overall size of the prototype and the intended indoor walking scenario. Based on previous leg-structure design experience and the requirement of maintaining sufficient leg flexibility, the coxa length was set to 75 mm, accounting for approximately 30% of the total leg length. The remaining length of 175 mm was then distributed between the femur and tibia. Six candidate femur-to-tibia ratios, namely 2:8, 3:7, 4:6, 1:1, 6:4, and 7:3, were evaluated in MATLAB R2023b (MathWorks, Natick, MA, USA) by calculating the reachable workspace of the foot tip. The corresponding segment lengths are listed in Table 1.

For each candidate ratio, the foot-tip reachable workspace was obtained from the forward kinematic model by sampling the joint variables within their allowable ranges. The evaluation mainly considered three workspace-related criteria. The first criterion was the forward reachable range, which affects the feasible stride length during straight-line walking. The second criterion was the vertical reachable range, which affects the foot-lifting capability during the swing phase. The third criterion was structural compactness, which affects the mechanical layout of the leg and helps avoid excessive distal extension or an overly folded configuration. These criteria are directly related to walking stability because insufficient forward reach restricts stride length, whereas insufficient vertical clearance increases the risk of early ground contact during the swing phase and may introduce additional body oscillation.

**Figure 2:**
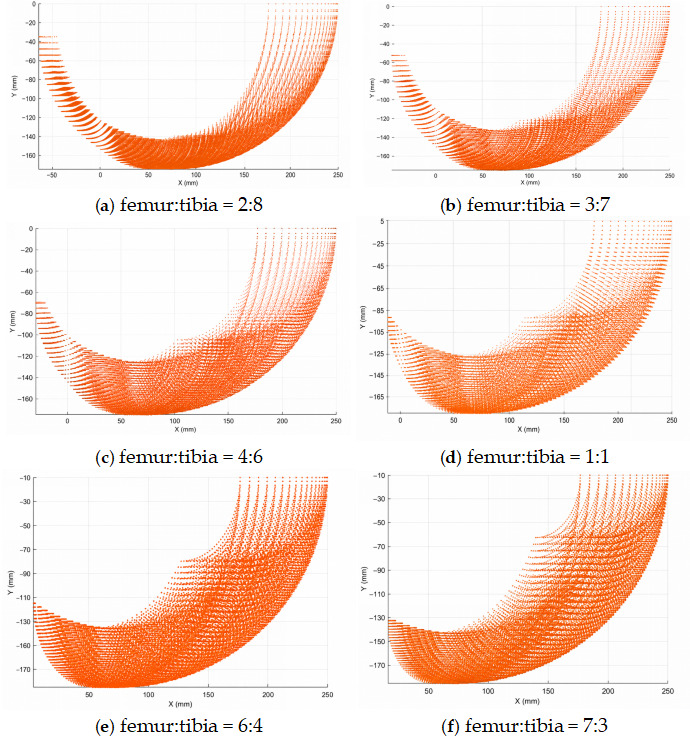
MATLAB-generated reachable workspace point-cloud distributions for candidate femur-to-tibia ratios under a fixed total leg length. (**a**) 2:8. (**b**) 3:7. (**c**) 4:6. (**d**) 1:1. (**e**) 6:4. (**f**) 7:3. The 4:6 ratio was selected as a balanced engineering compromise by considering forward reachable range, vertical foot-lifting capability, stride potential, and structural compactness.

The MATLAB-generated reachable workspace point-cloud distributions of the six candidate ratios are shown in Figure 2. When the femur was too short, as in the 2:8 and 3:7 ratios, the reachable workspace was mainly extended by the long tibia, but the proximal adjustment capability was limited. This configuration reduced the effective forward placement range of the foot and was not beneficial for achieving a stable stride. When the femur was too long, as in the 6:4 and 7:3 ratios, the tibia became relatively short, which compressed the distal workspace and weakened the foot-end adjustment capability during swing and touchdown. In particular, the 7:3 ratio produced an excessively short tibia, which limited the effective foot-placement region and made the leg less compact for the selected robot body size. The 1:1 ratio provided a moderate workspace, but it did not show a better balance between forward reach and vertical foot-lifting capability than the 4:6 ratio.

Based on this comparison, the 4:6 femur-to-tibia ratio was selected as a balanced engineering compromise rather than as a purely empirical or globally optimal choice. With this ratio, the femur and tibia lengths were set to 70 mm and 105 mm, respectively. This configuration provided a balanced reachable workspace for the selected stride length and lift height used in the subsequent tripod-gait experiments while maintaining a compact leg structure. Therefore, the selected 4:6 proportion was used in the following kinematic modeling, foot-trajectory planning, and walking-stability experiments.

### 2.4. Single-Leg Architecture and Compliant Foot-End

**Figure 3:**
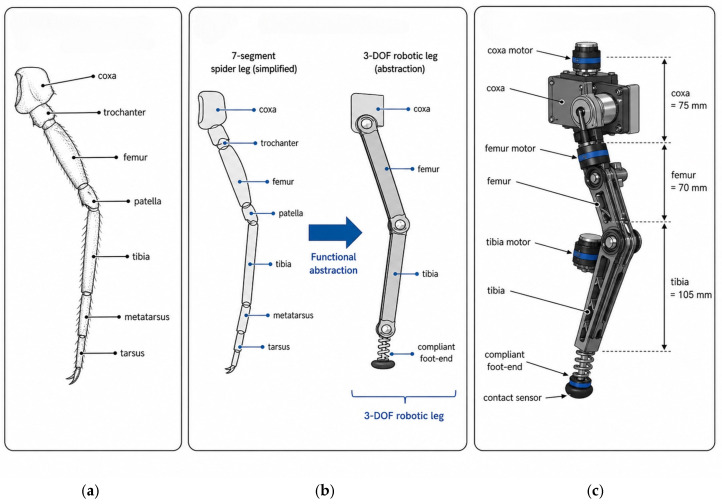
Biomimetic abstraction and engineering implementation of the spider-inspired 3-DOF leg. (**a**) Simplified schematic of the main biological segments of a spider leg. (**b**) Functional abstraction from seven biological leg segments to a compact three-degree-of-freedom robotic leg. (**c**) Structure of the implemented robotic leg, showing the coxa, femur, tibia, actuators, segment lengths, compliant foot-end module, and pressure/contact sensor.

The implemented single-leg architecture is shown in Figure 3c. Each leg consists of three serially connected structural segments: the coxa, femur, and tibia. The coxa, femur, and tibia lengths are 75 mm, 70 mm, and 105 mm, respectively. Three servo motors are used to actuate the leg, corresponding to one proximal yaw joint and two sagittal-plane joints. The proximal coxa motor adjusts the horizontal placement of the leg, while the femur and tibia motors control foot lifting, forward swing, and stance support. This 3-DOF configuration provides the basic motion capability required for tripod-gait walking while maintaining a compact mechanical structure.

Compared with a literal reproduction of spider anatomy, the simplified coxa–femur–tibia structure reduces the number of joints and actuators, thereby decreasing mechanical complexity and controller burden. At the same time, it preserves the functional characteristics required for stable walking, including leg placement, swing-phase foot lifting, and distal support during touchdown. The identical leg architecture was adopted for all six legs to simplify kinematic modeling and ensure consistent motion execution among the leg modules.

A compliant foot-end module was installed at the distal end of each leg to reduce touchdown impact. The module consists of a compact compression-spring mechanism and a pressure/contact sensor. The spring provides passive compliance during foot–ground contact, helping to buffer impact-induced vibration and reduce the transmission of shock to the robot body. The spring stiffness is approximately 2.9 N/mm, with a preload of 5.8 N. The pressure/contact sensor is used to detect the foot–ground contact state during gait execution.

In this study, the pressure/contact sensor was used mainly as a contact-state detection element rather than as a calibrated force-measurement system. Therefore, the walking-stability evaluation was based primarily on body-attitude angles and angular velocities instead of direct foot–ground force measurements. This clarification also avoids interpreting the sensor as an independent force-measurement device in the experimental results.

The compliant foot-end design improves the robustness of the leg during repeated swing–stance transitions. During the swing phase, the foot follows the planned trajectory generated by the gait controller. During touchdown, the compliant mechanism absorbs part of the contact impact, while the pressure/contact sensor provides foot–ground contact-state information for gait monitoring. This design is consistent with the biomimetic abstraction described in Section 2.1, in which the distal compliant contact of spider legs is simplified into an engineering foot-end support module.

## 3. Kinematic Modeling of the Hexapod Robot

### 3.1. Coordinate Frames and Forward Kinematics

**Figure 4:**
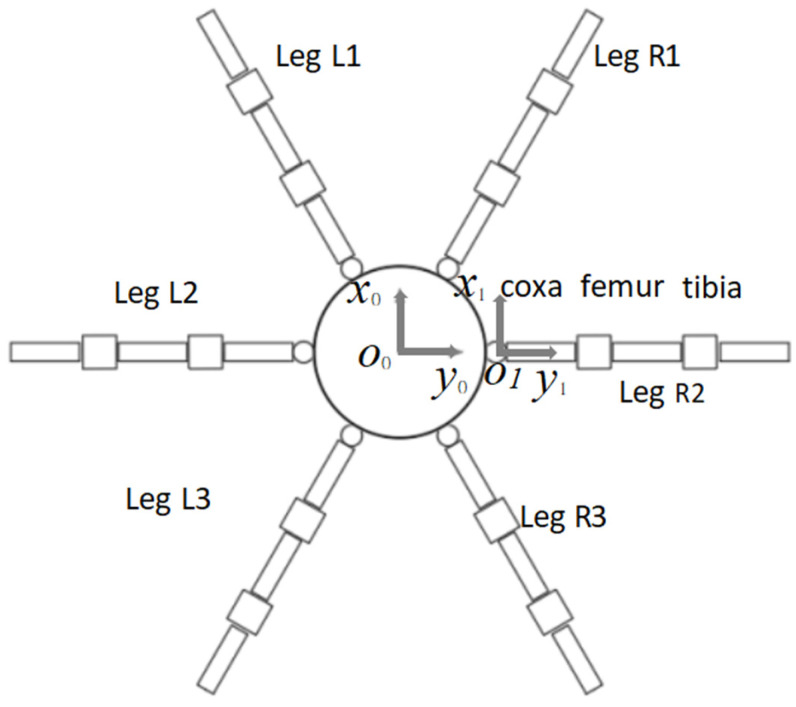
Body-fixed coordinate system, local leg coordinate system, and leg numbering of the hexapod robot. The central circular part represents the robot body, the six radially arranged structures represent the legs, and the arrows indicate the positive directions of the coordinate axes.

To describe the motion of the proposed hexapod robot, a body-fixed coordinate system was established at the geometric center of the robot body, and a local coordinate system was assigned to the coxa joint of each leg. The six legs were numbered as L1–L3 on the left side and R1–R3 on the right side. Because all six legs share the same mechanical configuration, the kinematic model of a single leg was first derived and then extended to the remaining legs according to their installation angles. The body-fixed coordinate system, local leg coordinate system, and leg numbering are shown in Figure 4.

**Figure 5:**
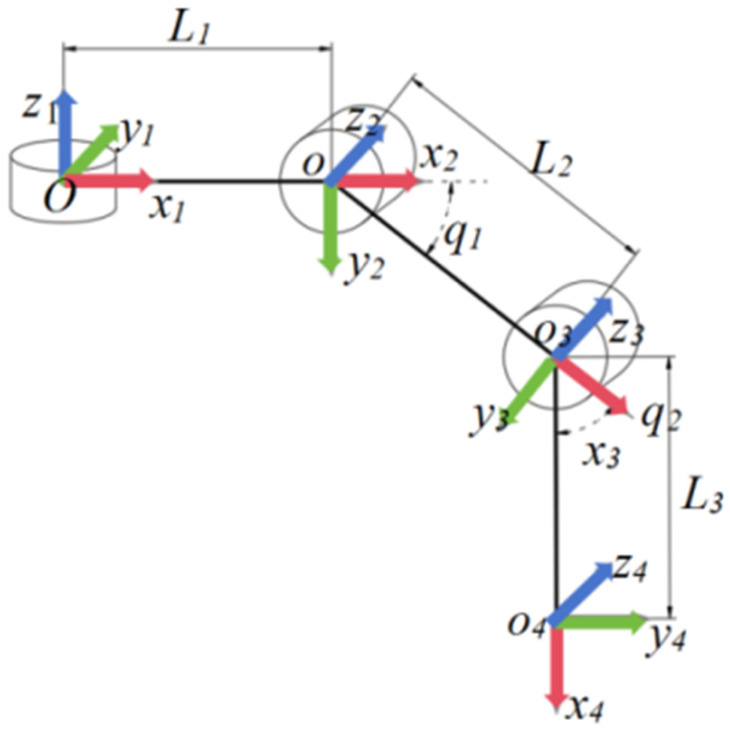
Local D–H coordinate system and joint variables q1, q2, and q3 of a single 3-DOF leg. The red, green, and blue arrows denote the positive directions of the x-, y-, and z-axes, respectively.

Each leg was modeled as a 3-DOF serial mechanism consisting of the coxa, femur, and tibia links. The three joint variables are denoted as q1, q2, and q3, corresponding to the coxa yaw joint, femur joint, and tibia joint, respectively. The link lengths of the coxa, femur, and tibia are denoted as L1, L2, and L3, respectively. In the implemented robot, L1 = 75 mm, L2 = 70 mm, and L3 = 105 mm. The local D–H coordinate system and joint variables of a single leg are shown in Figure 5.

According to the standard Denavit–Hartenberg (D-H) convention, the homogeneous transformation matrix between two adjacent joint coordinate systems can be expressed as

(1) $$\begin{array}{l} H_{\left(i+1\right)}^{i}=Rot(z,\theta_{i})\times Trans(z,d_{i})\times Trans(x,a_{i})\times Rot(x,\alpha_{i})\\ =\left[\begin{array}{cccc} \cos\theta_{i} & -\cos\alpha_{i}\sin\theta_{i} & \sin\alpha_{i}\sin\theta_{i} & a_{i}\cos\theta_{i}\\ \sin\theta_{i} & \cos\alpha_{i}\cos\theta_{i} & -\sin\alpha_{i}\cos\theta_{i} & a_{i}\sin\theta_{i}\\ 0 & \sin\alpha_{i} & \cos\alpha_{i} & d_{i}\\ 0 & 0 & 0 & 1 \end{array}\right] \end{array}$$

The individual transformation matrices of the coxa, femur, and tibia joints can be obtained from the D-H parameters. For concise presentation, only the overall transformation from the coxa coordinate system to the foot-end coordinate system is retained in the present paper:

(2) $$H_{4}^{1}=H_{2}^{1}(q_{1})\cdot H_{3}^{2}(q_{2})\cdot H_{4}^{3}(q_{3})$$

To express the foot-end position in the body-fixed coordinate system, the transformation from the foot-end coordinate system to the body-fixed coordinate system can be written as

(3) $$H_{4}^{0}=H_{1}^{0}\cdot H_{4}^{1}$$

where $H_{4}^{1}$ denotes the transformation from the local coxa coordinate system to the foot-end coordinate system, and $H_{1}^{0}$ denotes the transformation from the local leg coordinate system to the body-fixed coordinate system. The transformation from the local leg coordinate system to the body-fixed coordinate system is expressed as

(4) $$H_{1}^{0}=H(x,y,z)\cdot Rot(z,q_{0},i)\cdot \mathrm{Trans}(x,L_{b})$$

where H(x, y, z) represents the translational transformation of the body frame, $q_{0},i$ is the installation angle of the ith leg with respect to the body-fixed coordinate system, and $L_{b}$ is the distance from the body center to the corresponding coxa joint.

(5) $$\begin{array}{l} \left\{\begin{array}{l} x_{i}=\cos(q_{0},i)\cdot (L_{1}\cdot \cos(q_{1})+(L_{2}\cdot \cos(q_{2})+L_{3}\cdot \cos(q_{2}+q_{3}))\cdot \cos(q_{1}))\\ -\sin(q_{0},i)\cdot (L_{2}\cdot \cos(q_{2})+L_{3}\cdot \cos(q_{2}+q_{3}))\cdot \sin(q_{1})+L_{b}\cdot \cos(q_{0},i)\\ y_{i}=\sin(q_{0},i)\cdot (L_{1}\cdot \cos(q_{1})+(L_{2}\cdot \cos(q_{2})+L_{3}\cdot \cos(q_{2}+q_{3}))\cdot \cos(q_{1}))\\ +\cos(q_{0},i)\cdot (L_{2}\cdot \cos(q_{2})+L_{3}\cdot \cos(q_{2}+q_{3}))\cdot \sin(q_{1})+L_{b}\cdot \sin(q_{0},i)\\ z_{i}=L_{2}\cdot \sin(q_{2})+L_{3}\cdot \sin(q_{2}+q_{3}) \end{array}\right. \end{array}$$

where $x_{i}$, $y_{i}$, and $z_{i}$ are the foot-end coordinates of the i-th leg in the body-fixed coordinate system. The above equations establish the forward kinematic mapping between the joint space and the foot-end workspace, providing the basis for subsequent inverse kinematics, workspace analysis, tripod-gait planning, and Bézier foot-trajectory generation.

### 3.2. Inverse Kinematics

Inverse kinematics was used to calculate the joint variables of each leg from the desired foot-end position. In the proposed robot, the planned foot-end trajectory is first generated in Cartesian space and then converted into the corresponding joint-angle commands for the coxa, femur, and tibia motors. Therefore, the inverse kinematic model provides the direct connection between foot-end trajectory planning and servo-level motion control.

Based on the forward kinematic model established in Section 3.1, the desired foot-end position of the i-th leg in the body-fixed coordinate system is denoted as P = [P_x_, P_y_, P_z_]^T^. Considering the installation angle of the i-th leg, $q_{0},i$, and the distance from the body center to the corresponding coxa joint, $L_{b}$_,_ the auxiliary geometric variables are defined as follows:

(6) $$\left\{\begin{array}{l} r=\sqrt{P_{x}^{2}+P_{y}^{2}}\\ r_{l}=\sqrt{{\left(P_{x}-L_{b}\sin\left(q_{0},i\right)\right)}^{2}+{\left(P_{y}-L_{b}\cos\left(q_{0},i\right)\right)}^{2}}\\ d=\sqrt{r_{l}^{2}+P_{Z}^{2}} \end{array}\right.$$

where r is the horizontal distance from the body center to the desired foot-end point, $r_{l}$ is the horizontal distance from the local leg base to the desired foot-end point, and d is the distance from the femur joint to the desired foot-end point in the sagittal plane. According to the geometric relationship among the coxa, femur, tibia, and the desired foot-end point, the inverse kinematic relationship can be expressed as follows:

(7) $$\left\{\begin{array}{l} q_{1}=\arctan\left(\frac{P_{y}-L_{b}\cos\left(q_{0},i\right)}{P_{x}-L_{b}\sin\left(q_{0},i\right)}\right)\\ q_{2}=arccos\left(\frac{d^{2}-L_{2}^{2}-L_{3}^{2}}{2L_{2}L_{3}}\right)\\ q_{3}=arctan\left(\frac{P_{z}}{r_{l}-L_{2}+L_{3}\cos q_{2}}\right) \end{array}\right.$$

where q_1_, q_2_, and q_3_ denote the coxa, femur, and tibia joint variables, respectively; L_2_ and L_3_ are the femur and tibia lengths, respectively; $L_{b}$ is the distance from the body center to the corresponding coxa joint; and $q_{0},i$ is the installation angle of the i-th leg.

In practical gait execution, the desired foot-end positions generated by the Bézier and composite cycloid trajectories are substituted into the inverse kinematic equations to obtain the corresponding joint-angle commands. The calculated joint variables are further adjusted according to the installation direction and zero-position offset of each servo motor before being sent to the robot controller. This inverse kinematic solution enables the Cartesian foot-end trajectories to be executed by the physical hexapod robot during tripod-gait walking.

## 4. Hierarchical Motion Control Architecture

**Figure 6:**
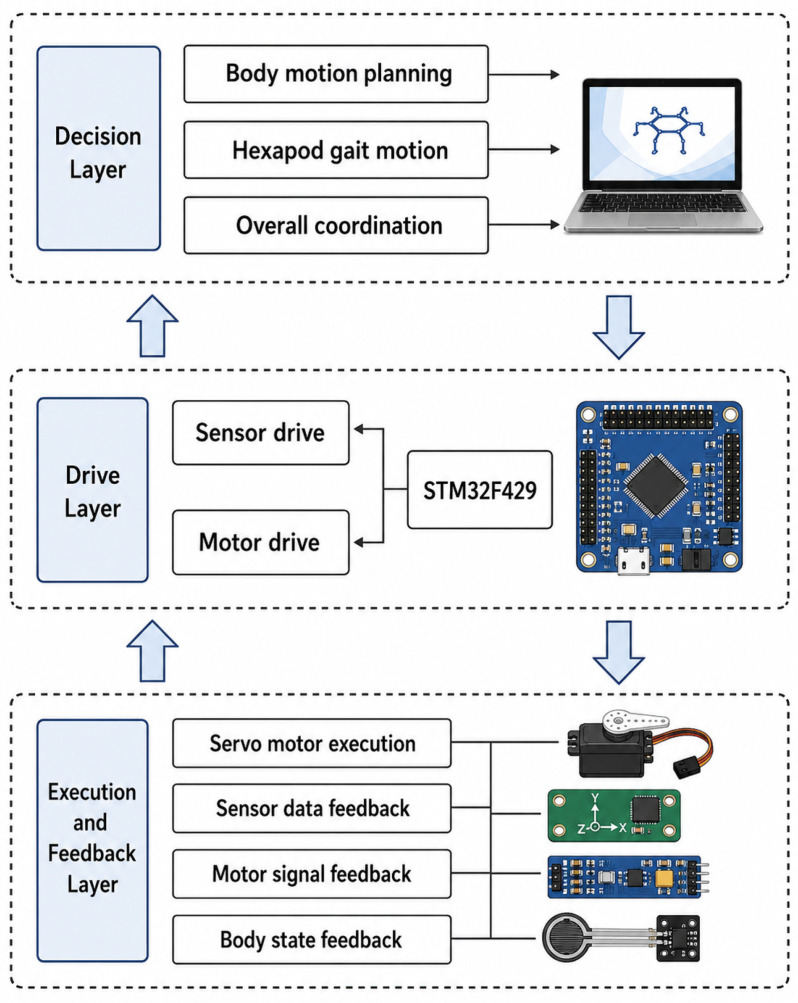
Hierarchical motion control architecture of the hexapod robot, including the decision layer, drive layer, and execution and feedback layer. The downward arrows indicate the transmission of motion-planning commands from the decision layer to the lower execution units, whereas the upward arrows indicate feedback information returned from the sensors and actuators to the upper layers.

To implement gait planning and coordinated locomotion of the proposed hexapod robot, a hierarchical motion control architecture was adopted, as shown in Figure 6. The control system consists of three functional layers: the decision layer, the drive layer, and the execution and feedback layer. The decision layer is responsible for high-level motion planning and gait-trajectory generation. The drive layer receives the planned motion parameters and converts them into executable joint-control commands. The execution and feedback layer carries out low-level actuator execution and collects feedback information from the motors and onboard sensors. This layered architecture separates high-level planning, embedded command distribution, and low-level execution and feedback, thereby improving the modularity and maintainability of the overall control system.

### 4.1. Decision Layer

The decision layer is responsible for high-level data processing and motion-planning algorithm execution. According to task instructions and user-defined motion parameters, this layer performs body motion planning, gait generation, and foot-end trajectory planning. The resulting motion parameters include gait sequence, stride length, lift height, gait period, and desired foot-end trajectory points. Because these processes involve relatively high computational cost, a personal computer (PC) is used as the upper-level controller. The generated trajectory and gait parameters are then transmitted to the drive layer through the communication interface.

### 4.2. Drive Layer

The drive layer serves as the intermediate control layer between the upper-level planner and the low-level execution units. It receives the motion parameters generated by the decision layer and converts the planned foot-end trajectories into joint-control commands through inverse kinematic calculation. In the present system, an STM32F429 microcontroller (STMicroelectronics, Geneva, Switzerland) is used for embedded command processing, signal distribution, and communication with the servo motors and onboard sensors. This layer ensures that the motion commands of the six legs are updated in a coordinated manner during tripod-gait walking.

### 4.3. Execution and Feedback Layer

The execution and feedback layer is responsible for actuator execution and feedback acquisition. It receives joint-control commands from the drive layer and sends the corresponding control signals to the servo motors. At the same time, feedback signals from the motors and onboard sensors are collected and returned to the upper layers for state monitoring and motion adjustment. These feedback signals include motor state information, sensor data, and body-attitude information. This closed-loop information flow helps monitor the execution of the planned gait and supports stable locomotion of the hexapod robot.

## 5. Tripod Gait Design of the Hexapod Robot

To achieve stable forward walking, the proposed hexapod robot adopts an alternating tripod gait. In this gait pattern, three legs remain in the support phase while the other three legs perform swing motion. The six legs are divided into two tripod groups: group A consists of L1, L3, and R2, while group B consists of L2, R1, and R3. During locomotion, these two groups alternate between the support phase and the swing phase. This gait provides a practical balance between walking efficiency and locomotion stability during straight-line motion.

**Figure 7:**
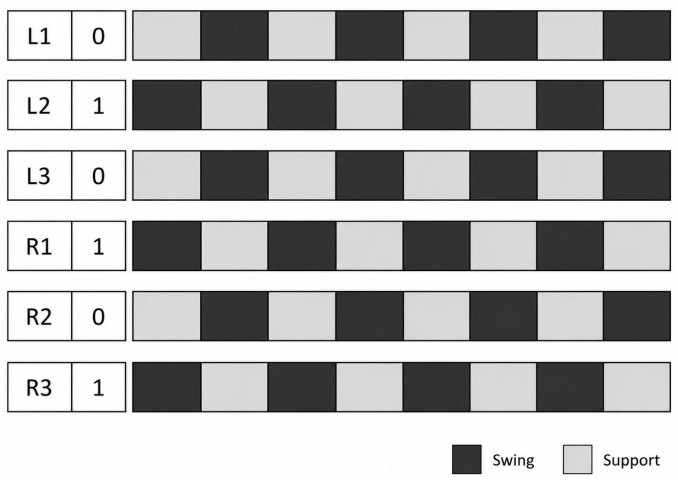
Gait sequence diagram of the alternating tripod gait adopted by the hexapod robot. Dark blocks and light blocks denote the swing and support phases, respectively.

The locomotion cycle of the robot is divided into two alternating phases. In the first phase, group A supports the robot body and propels it forward, while group B is lifted from the ground and swings toward the next foothold. In the second phase, group B becomes the supporting tripod after touchdown, while group A is lifted and enters the swing phase. Through the periodic alternation of these two phases, the robot achieves continuous forward locomotion. The gait sequence diagram is shown in Figure 7.

In Figure 7, “0” denotes the initial support phase, and “1” denotes the initial swing phase. The dark blocks indicate swing motion, whereas the light blocks indicate support motion. In the initial state, legs L1, L3, and R2 form the supporting tripod, while legs L2, R1, and R3 form the swinging tripod. After the swinging legs complete one step and touchdown, they transition into the support phase, while the previous supporting tripod is lifted into the swing phase. Repetition of this alternating process completes one full gait cycle of the robot.

**Figure 8:**
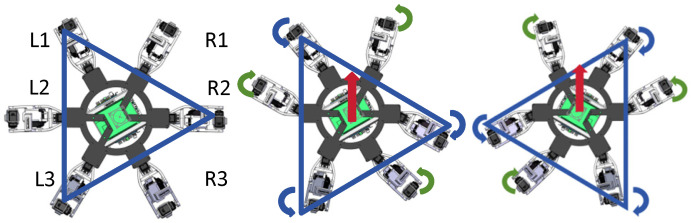
Schematic illustration of alternating tripod support during straight-line walking. The blue triangle indicates the support polygon formed by the three supporting legs, the red arrow indicates the forward walking direction, and the colored curved arrows indicate the leg swing directions during tripod gait. The triangular support formed by three legs alternates between the two tripod groups during locomotion.

The corresponding support relationship during straight-line walking is illustrated in Figure 8. The alternating tripod support pattern ensures that three legs remain in contact with the ground during each support phase, thereby maintaining stable body support and coordinated forward motion.

## 6. Bézier Foot-End Trajectory Planning

Foot-end trajectory design plays an important role in the walking performance of legged robots because it directly affects swing smoothness, foot clearance, touchdown transition, foot–ground impact, and body-motion stability [23]. In conventional hexapod gait planning, the composite cycloid trajectory is widely used because it provides adjustable stride length and leg-lift height with a relatively simple mathematical form. Therefore, the composite cycloid trajectory was used as the baseline trajectory for comparison in this study. It is expressed as

(8) $$x=S\left(\frac{t}{T}-\frac{1}{2\pi }\sin\left(2\pi \frac{t}{T}\right)\right)$$

(9) $$z=H\left(\frac{1}{2}-\frac{1}{2}\cos2\pi \frac{t}{T}\right)$$

where T denotes the swing-phase period, S is the stride-related parameter, H is the maximum leg-lift height, and t is the time variable during the swing phase.

Although the composite cycloid trajectory is simple and practical, its trajectory shape is mainly determined by the stride length and lift height. Therefore, it provides limited flexibility for independently adjusting the lift-off region, mid-swing foot clearance, and touchdown transition. In particular, relatively abrupt acceleration variation near lift-off and touchdown may increase foot–ground impact and body oscillation during walking. To obtain a smoother and more adjustable swing trajectory, an eleventh-order Bézier curve was introduced for foot-end trajectory planning.

The general Bézier expression is given by

(10) $$B\left(t\right)=\sum_{i=0}^{n}\left(\begin{array}{c} n\\ i \end{array}\right)P_{i}{\left(1-t\right)}^{n-i}t^{i},t\in \left[0,1\right]$$

where $P_{i}$ denotes the *i*-th control point, *n* is the curve order, $\left(\begin{array}{c} n\\ i \end{array}\right)$ is the binomial coefficient, and t∈[0,1] is the normalized curve parameter.

An *n*-th-order Bézier curve contains *n* + 1 control points. The first and last control points define the start and end of the trajectory, whereas the intermediate control points determine the geometric shape, local smoothness, and foot-clearance characteristics of the swing path. In this study, an eleventh-order Bézier curve was adopted because it provides twelve control points, allowing the swing trajectory to be divided into lift-off, mid-swing, and touchdown regions with sufficient local shape-adjustment flexibility. Compared with lower-order Bézier curves, the eleventh-order form provides more control points to regulate the start and end regions of the trajectory independently while maintaining a smooth swing curve. The objective was not to prove that the eleventh order is globally optimal, but to obtain an engineering trajectory with sufficient smoothness and adjustability for comparison with the composite cycloid trajectory under identical tripod-gait conditions.

The stride length and lift height were selected according to the reachable workspace of the proposed leg and the requirement of maintaining sufficient foot clearance during flat-ground tripod-gait walking. The selected stride length was kept within the effective forward reachable workspace of the 4:6 femur-to-tibia leg proportion, while the lift height was chosen to provide adequate ground clearance without causing excessive body oscillation. In the subsequent walking experiments, the forward stride length was set to 100 mm, the maximum leg-lift height was set to 50 mm, and the swing and support phases were both set to 1 s.

The control points used in the proposed eleventh-order Bézier trajectory are listed in Table 2. Here, S denotes the half-stride parameter in the symmetric Bézier trajectory definition, so 2S corresponds to the 100 mm forward stride used in the walking experiments, and H denotes the leg-lift height.

**Figure 9:**
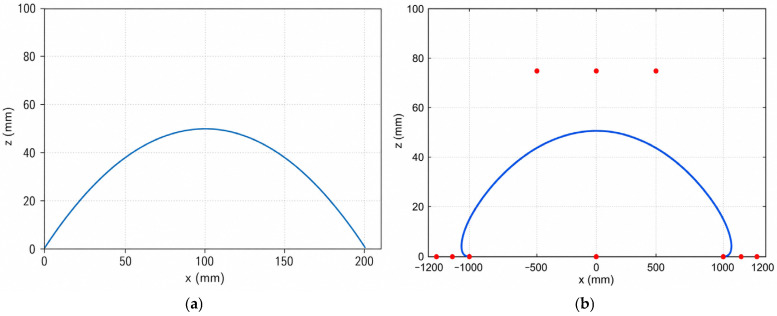
Comparison of foot-end swing trajectories generated in MATLAB under the same gait parameters. (**a**) Composite cycloid trajectory. (**b**) Eleventh-order Bézier trajectory. The forward stride length was 100 mm, and the maximum leg-lift height was 50 mm.

**Figure 10:**
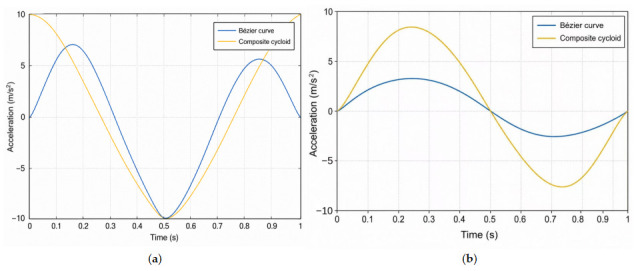
Comparison of foot-end acceleration profiles under the proposed Bézier trajectory and the conventional composite cycloid trajectory: (**a**) z-axis acceleration; (**b**) x-axis acceleration.

The composite cycloid trajectory and the eleventh-order Bézier trajectory generated in MATLAB under the same gait parameters are shown in Figure 9. For both trajectories, the forward stride length was set to 100 mm and the maximum leg-lift height was set to 50 mm. The acceleration profiles of the two trajectories are compared in Figure 10. The results show that the Bézier trajectory provides smoother acceleration variation near lift-off and touchdown, especially in the vertical direction. Compared with the composite cycloid trajectory, the acceleration of the Bézier trajectory approaches zero more smoothly at the beginning and end of the swing phase, indicating a smoother transition between the swing phase and ground contact. This characteristic helps reduce landing impact and suppress body-motion disturbance during walking.

Compared with the conventional composite cycloid method, the proposed Bézier-based trajectory provides greater flexibility in shaping the swing path through control-point adjustment while maintaining continuous position, velocity, and acceleration evolution. Therefore, it is suitable for stable tripod-gait walking of the proposed hexapod robot under the tested flat-ground conditions.

## 7. Experimental Validation of Walking Stability

**Figure 11:**
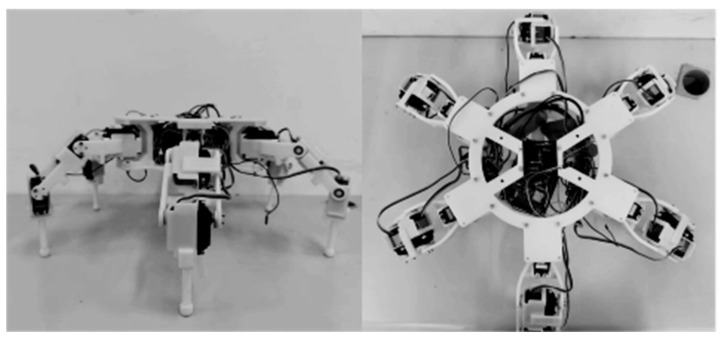
Prototype of the hexapod robot used in the walking experiment.

To evaluate the effectiveness of the proposed Bézier foot-end trajectory, straight-line walking experiments were conducted using the prototype hexapod robot. The robot prototype is shown in Figure 11. The main body was fabricated from carbon-fiber plates, while the feet were primarily made of acrylonitrile butadiene styrene (ABS). The experiments were carried out on a flat surface to compare the walking stability of the proposed eleventh-order Bézier trajectory with that of the conventional composite cycloid trajectory under identical tripod-gait conditions.

**Figure 12:**
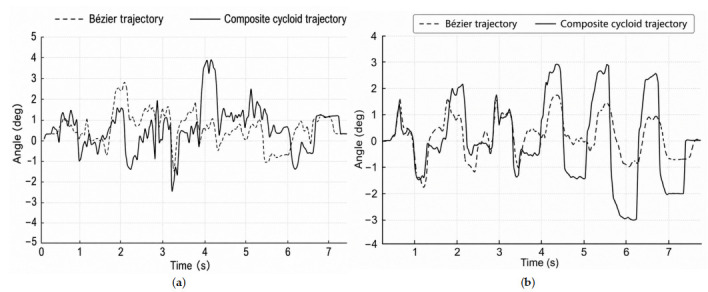
Body-attitude angle variations during straight-line walking under the Bézier and composite cycloid trajectories: (**a**) pitch-angle variation; (**b**) roll-angle variation.

**Figure 13:**
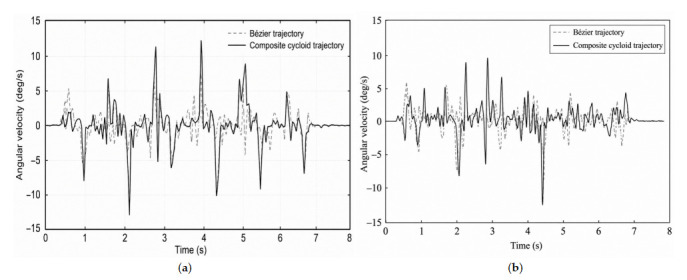
Body angular-velocity variations during straight-line walking under the Bézier and composite cycloid trajectories: (**a**) pitch angular-velocity variation; (**b**) roll angular-velocity variation.

The walking experiments were conducted using the same gait parameters described in Section 6. During straight-line walking, body-attitude angles and angular velocities were recorded by the onboard inertial measurement unit. The pitch angle, roll angle, pitch angular velocity, and roll angular velocity were used as stability-related indicators. The corresponding results are shown in Figure 12 and Figure 13.

As shown in Figure 12, the Bézier-based trajectory yielded smaller body-attitude fluctuations than the composite cycloid trajectory. Under the Bézier trajectory, the pitch and roll angles remained within a narrower fluctuation range and exhibited smoother periodic variation. In contrast, the composite cycloid trajectory produced larger attitude oscillations, especially during the swing–support transition.

Figure 13 further shows the pitch and roll angular-velocity variations during straight-line walking. The angular-velocity curves under the Bézier trajectory were generally smoother and showed fewer abrupt peaks than those under the composite cycloid trajectory. This indicates that the Bézier trajectory reduced sudden body-motion changes during foot lift-off and touchdown, which is consistent with the smoother acceleration profile discussed in Section 6.

Overall, the experimental results demonstrate that the proposed eleventh-order Bézier foot-end trajectory can improve the flat-ground walking stability of the hexapod robot under the tested conditions. However, the present validation was limited to straight-line walking on a flat surface, and further experiments on uneven terrain, slopes, and obstacle-crossing scenarios are still needed to evaluate the robustness of the proposed trajectory in more complex environments.

## 8. Conclusions

In this study, a spider-leg-inspired hexapod robot was developed, and its structural design, kinematic modeling, tripod gait pattern, and foot-end trajectory planning were investigated. Selected functional characteristics of spider legs were abstracted into a simplified 3-DOF leg structure rather than directly reproducing spider anatomy. A workspace-based leg-proportion analysis was conducted under a fixed total leg length, and a 4:6 femur-to-tibia ratio was selected as a balanced engineering compromise by considering forward reach, vertical foot-lifting capability, stride potential, and structural compactness.

To improve swing-phase smoothness, an eleventh-order Bézier foot-end trajectory was introduced and compared with a conventional composite cycloid trajectory under identical tripod-gait conditions. The Bézier trajectory provided greater flexibility in shaping the lift-off, mid-swing, and touchdown regions through control-point adjustment. The acceleration comparison indicated that the Bézier trajectory produced smoother transition characteristics near lift-off and touchdown, which is beneficial for reducing foot–ground impact and body-motion disturbance.

Straight-line walking experiments on a flat surface further showed that the Bézier-based trajectory reduced body-attitude fluctuation and produced smoother angular-velocity variation compared with the composite cycloid trajectory. These results indicate that the proposed structural design and Bézier-based foot-end trajectory can improve the flat-ground walking stability of the hexapod robot under the tested conditions.

The present study provides a practical reference for biomimetic leg-structure design and gait-trajectory planning of hexapod robots. However, the experimental validation was limited to straight-line walking on flat ground. Future work will focus on evaluating the proposed method under more complex terrain conditions, including uneven surfaces, slopes, and obstacle-crossing scenarios, and on further improving adaptive gait control for unstructured environments.

## Figures and Tables

**Table 1:** Candidate femur-to-tibia ratios used in the workspace-based leg-proportion analysis.

Femur-to-Tibia Ratio	Femur Length (mm)	Tibia Length (mm)	Main Workspace Characteristic
2:8	35.0	140.0	Limited forward reach and reduced effective stride potential
3:7	52.5	122.5	Improved reach compared with 2:8, but still limited horizontal workspace
4:6	70.0	105.0	Balanced horizontal reach, vertical foot-lifting capability, and compactness
1:1	87.5	87.5	Moderate workspace, but no clear advantage over 4:6
6:4	105.0	70.0	Reduced distal adjustment capability and compressed vertical workspace
7:3	122.5	52.5	Excessively short tibia, limited effective foot-placement region, and poor compactness

**Table 2:** Control points of the eleventh-order Bézier trajectory.

P_i_	X_i_	Z_i_
0	−S	0
1	−1.1S	0
2	−1.2S	0
3	−1.3S	0
4	−0.5S	1.5H
5	0	1.5H

## Data Availability

The data presented in this study are available from the corresponding author upon reasonable request.

## Abbreviations

ABS	acrylonitrile butadiene styrene
D-H	Denavit–Hartenberg
3-DOF	three degrees of freedom
PC	personal computer
